# From bedside to portable and wearable: development of a conformable ultrasound patch for deep breast tissue imaging

**DOI:** 10.1002/1878-0261.13531

**Published:** 2023-10-03

**Authors:** Mark T. Burgess, Jill Gluskin, Katja Pinker

**Affiliations:** ^1^ Department of Medical Physics Memorial Sloan Kettering Cancer Center New York NY USA; ^2^ Department of Radiology, Breast Imaging Service Memorial Sloan Kettering Cancer Center New York NY USA

**Keywords:** breast cancer, piezoelectrics, ultrasound imaging, wearable electronics

## Abstract

A breakthrough study from Du et al. has developed a wearable, ultrasound imaging patch for standardized and reproducible breast tissue imaging. The technology utilizes a honeycomb patch design to facilitate guided movement of the ultrasound array, enabling comprehensive, multiangle breast imaging. The system was validated *in vitro* and *in vivo* with a single human subject and has the potential for early‐stage breast cancer detection. This study addressed the current limitations of wearable ultrasound technologies, including imaging over large, curvilinear organs and integration of superior piezoelectric materials for high‐performance ultrasound arrays. The transition of ultrasound from the bedside to portable and wearable devices will pave the way for integration with big data collection, such as artificial intelligence (AI)‐based diagnosis and personalized ultrasonographic profile generation, for rapid and objective measurements. This advancement is especially important in the context of breast cancer, where early diagnosis and assessment of medical therapy responses are paramount to patient care.

## Graphical abstract

Wearable ultrasound technology is gaining interest for on‐demand medical diagnosis. While ultrasound is widely used for breast imaging, capturing large‐area curvilinear organs poses a challenge for wearable technologies. Recently, a study by Du et al. addressed this by introducing a wearable, conformable ultrasound breast patch. This technology could complement artificial intelligence (AI)‐driven ultrasound, thereby enhancing the accuracy of breast cancer diagnosis.

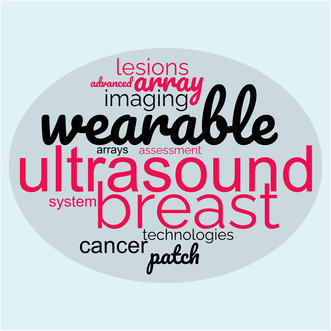

**Abbreviations**
ABUS: automated breast ultrasound
AI: artificial intelligence
BI‐RADS: breast imaging report and data system
cUSBr‐Patch: conformable ultrasound breast patch
HHUS: handheld ultrasound
MRI: magnetic resonance imaging
QUS: quantitative ultrasound
WBUT: whole breast ultrasound tomography

Handheld ultrasonography (HHUS) has long served as a valuable supplement to mammography for breast cancer screening, particularly in women with dense breasts where cancers are often mammographically occult [1]. Handheld ultrasonography (HHUS), because of its efficacy, safety, and low cost, is often used as a complementary breast imaging modality for the assessment of abnormal findings during screening mammography, breast magnetic resonance imaging (MRI), clinical breast exams, image‐guided interventions, and staging. Ultrasound can assess lesions in both predominately fatty breasts and dense, glandular structures and classify lesions based on the Breast Imaging Report and Data System (BI‐RADS) [2]. Ultrasound can evaluate the shape, orientation, internal structure, and margins of lesions to aid in the differentiation between benign and malignant tumors. A high level of skill is needed to obtain good images with HHUS. Sonographers must understand access windows, the differences in anatomy, and the numerous planes of view. Other ultrasound breast imaging technologies, such as automated breast ultrasound (ABUS) and whole breast ultrasound tomography (WBUT), can overcome some of the limitations of HHUS. A concern for screening ultrasound has traditionally been that it had high recall rates ranging from 10% to 30%; recent publications have shown more promising results with ABUS with recall rates below 2.5% while retaining good sensitivity [3, 4]. However, automated methods still rely on skin contact with a liquid medium and bulky machines in a hospital setting [5, 6].

Piezoelectric biomaterials have been developed to push ultrasound technologies from the bedside setting to wearable electronics [7]. Over the past 5 years, there has been a rapid emergence of piezoelectric‐based conformable ultrasound electronics for applications such as continuous cardiac imaging [8], blood flow monitoring [9], and imaging of other internal organs [10]. While these technologies are promising, there are challenges to using traditional piezoelectric‐based ultrasound transducers that are not easily conformable to curved body surfaces. New materials and advanced microfabrication techniques are also needed to avoid the toxicity of conventional lead‐based piezoelectric materials.

Recently, an engineering research article published in *Science Advances* has pushed the boundaries of ultrasound technology by developing a breast tissue scanning and imaging patch [11]. The wearable, conformable ultrasound breast patch (cUSBr‐Patch) is intended to eliminate operator training and apply transducer compression to provide standardized and reproducible image acquisition over the entire breast. This would put less reliance on the operator and facilitate a quicker and more objective analysis of breast tissue. Three main components comprised the cUSBr‐Patch: (a) soft fabric bra, (b) plastic honeycomb patch to hold and guide movement of the ultrasound array, and (c) tracker attached to the ultrasound array to denote position and rotate the array at a given location. The patch is attached to the fabric bra using magnets and circular holes were cut in the bra to align with the patch, which enables the array to be in direct contact with the skin. A tracker was used to traverse up to 15 hexagonal sections through the patch for a comprehensive set of overlapping ultrasound images that cover all relevant areas of the breast.

Diced [Yb/Bi‐Pb(In_1/2_Nb_1/2_)O_3_‐Pb(Mg_1/3_Nb_2/3_)O_3_‐PbTiO_3_] (Yb/Bi‐PIN‐PMN‐PT) single crystals were determined to be the most suitable array material due to their promising piezoelectric and dielectric properties for high‐voltage and high‐frequency medical transducer arrays. The arrays were fabricated and tested using traditional techniques, including a planar ultrasound phantom with wire and cylindrical targets to demonstrate field of view and resolution. The 7.0 MHz array had a maximum field of view of up to 10 cm width with an imaging depth of 8 cm. Most importantly, an *in vivo* study was carried out using a female subject with a history of breast anomalies. Both breasts were imaged with the cUSBr‐patch and cross‐validated with a commercial ultrasound system. Results showed that the cUSBr‐patch was able to identify a larger cyst in the left breast (1 cm diameter) and a smaller cyst (0.3 cm diameter) in the right breast. The field of view of the cUSBr‐patch was comparable to the commercial ultrasound array, although was unable to cover some shallow places closer to the skin. The researchers detected the larger cyst with similar image quality over 30‐min scanning window, consisting of 15‐min intervals, and confirmed the reproducibility of the array positioning. The authors comment that future research will focus on numerous advancements, such as embedding multiple arrays into a size‐customizable path to avoid random scanning, additional clinical trials during the medical treatments, and moving toward a portable system with back‐end capabilities to enable daily self‐screening. This could permit integration with big data collection for fast and objective measurements.

Ultrasound technologies that supplement standard B‐mode could aid the cUSBr‐patch in classifying breast lesions using BI‐RADS, which is typically limited to qualitative assessment with B‐mode alone [12]. Conventional Doppler ultrasound imaging has a limited role in characterizing the vascular architecture of breast tumors, which in the case of malignant tumors can present a chaotic distribution and irregular branches. Recently, more advanced Doppler methods have been implemented to increase microvascular flow sensitivity, thereby improving breast tumor classification [13]. In addition, quantitative ultrasound (QUS) analyses of the underlying radiofrequency ultrasound data are promising for multiparametric classification of breast lesions [14]. Taken together, combining additional modes like elastography and contrast‐enhanced ultrasound imaging with artificial intelligence (AI)‐enhanced ultrasound could improve clinical workflows, ultrasound‐based diagnosis, and characterization of breast lesions.

Breast cancer is the most frequently diagnosed cancer globally with over 2.3 million new cases and 685 000 deaths annually, as reported in 2020 [15]. While most cases occur in transitioned countries, a disproportionate share of breast cancer deaths occur in emerging countries with limited access to advanced imaging modalities such as MRI for lesion characterization. Early diagnosis of breast cancer is important for its treatment and prognosis; therefore, accurate lesion assessment is critical in reducing the number of deaths arising from it. The study from Du et al. puts forth a prototype system that could enable more accessible and reproducible breast imaging without the need for advanced operator training. Combining this technology with AI‐enhanced ultrasound could help combat the disproportionate death rate of breast cancer in developing countries.

## Conflict of interest

The authors declare no conflict of interest.
